# Bioadhesive Surfactant Systems for Methotrexate Skin Delivery

**DOI:** 10.3390/molecules21020231

**Published:** 2016-02-18

**Authors:** Giovana Aparecida de Souza Cintra, Larissa Alvarenga Pinto, Giovana Maria Fioramonti Calixto, Christiane Pienna Soares, Eliete de Souza Von Zuben, Maria Virgínia Scarpa, Maria Palmira Daflon Gremião, Marlus Chorilli

**Affiliations:** 1 Faculdade de Ciências Farmacêuticas, UNESP—Universidade Estadual Paulista, Campus Araraquara, Departamento de Fármacos e Medicamentos, Araraquara, SP, 14800-850, Brazil; giovanacintra@gmail.com (G.A.S.C.); larissa_alvarenga@ymail.com (L.A.P.); eliete.vz@gmail.com (E.S.V.Z.); scarpamv@fcfar.unesp.br (M.V.S.); pgremiao@fcfar.unesp.br (M.P.D.G.); 2 Faculdade de Ciências Farmacêuticas, UNESP—Universidade Estadual Paulista, Campus Araraquara, Departamento de Análises Clínicas, Araraquara, SP 14800-850, Brazil

**Keywords:** microemulsion, liquid-crystalline systems, *in vitro* skin permeation, *in vitro* skin retention, polyether functional siloxane, methotrexate

## Abstract

Methotrexate (MTX) is an immunosuppressive drug for systemic use in the treatment of skin diseases, however, MTX presents a number of side effects, such as hepatotoxicity. To overcome this limitation, this study developed skin MTX delivery surfactant systems, such as a microemulsion (ME) and a liquid crystalline system (LCS), consisting of a glycol copolymer-based silicone fluid (SFGC) as oil phase, polyether functional siloxane (PFS) as surfactant, and carbomer homopolymer type A (C971) dispersion at 0.5% (*wt*/*wt*) as aqueous phase. Polarized light microscopy and small-angle X-ray scattering evidenced the presence of hexagonal and lamellar LCSs, and also a ME. Texture profile and *in vitro* bioadhesion assays showed that these formulations are suitable for topical application, showing interesting hardness, adhesiveness and compressibility values. Rheology analysis confirmed the Newtonian behaviour of the ME, whereas lamellar and hexagonal LCSs behave as pseudoplastic and dilatant non-Newtonian fluids, respectively. *In vitro* release profiles indicated that MTX could be released in a controlled manner from all the systems, and the Weibull model showed the highest adjusted coefficient of determination. Finally, the formulations were not cytotoxic to the immortalized human keratinocyte line HaCaT. Therefore, these bioadhesive surfactant systems established with PFS and C971 have great potential as skin delivery systems.

## 1. Introduction

**Figure 1:**
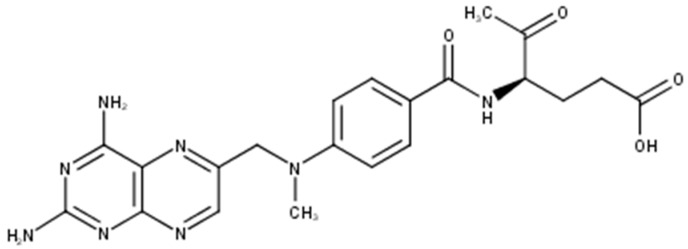
Molecular structure of methotrexate.

Methotrexate (MTX), also known as amethopterin whose structure is shown in Figure 1, is a cytotoxic antifolate drug approved by U.S. Food and Drug Administration (FDA) as an anti-psoriatic agent since 1971 [1]. It is one of the most prominent substances used systemically in the treatment of psoriasis and it is used also in cancer treatment as a cytostatic agent. It competitively inhibits the activity of the enzyme dihydrofolate reductase, an enzyme necessary for the synthesis of nucleotides and amino acids, which reduces DNA synthesis, inhibits mitosis and proliferation of rapidly dividing cells, such as the epidermis, and bone marrow [2,3,4].

Visible skin diseases are correlated with increased rates of depression, anxiety and low self-esteem in patients. Because of that, medical treatment for skin conditions complements the medical therapy of psychological health. Considering the reduction in quality of life and the psychosocial disability suffered by patients, there is the need for prompt, effective treatment and long-term disease control [5].

Considering the important potential side events of the MTX therapy such as, hepatotoxicity, myelosuppression, and pulmonary fibrosis, robust guidelines have been developed in order to monitory the treatment with this drug [6]. Furthermore, cutaneous ulceration has been described as an early clinical sign of impending systemic toxicity [7].

Therefore, a strategic way to overcome this toxicity barrier is the development of novel MTX-loaded skin delivery for the MTX absorption into and across skin, by passive drug diffusion through the lipid domains of stratum corneum (governed by difference in the drug concentration between drug delivery systems and blood); thus, this results in the optimum dosage for long periods, increase the efficacy of drug and, thus, increase patient compliance [8]. 

Among these systems, it has been shown the interest in the development of surfactant systems, such as liquid crystalline systems (LCSs) and microemulsions (MEs) for topical drug delivery. Since surfactants have the ability to stabilize components with different polarities, additional regions for the solubilization of hydrophilic and lipophilic drugs are created [9,10].

Application of the surfactant polyether functional siloxane in LCSs and MEs has been studied in our research group because of its non-irritant properties and its ability to stabilize drug delivery systems [11]. These systems exhibit many advantages, such as the fact that the promote controlled release of drugs and protect active principles from thermal or photodegradation [12,13]. Consequently, finding a system that meets these requirements would be extremely valuable.

Considering the intended skin application of these systems, the bioadhesion of the formulations is an important factor that should be improved. One of the ways of achieving this is through the incorporation of Carbopol^®^ polymers in these systems [10,14,15] These polymers are very high molecular weight polymers of acrylic acid (PAA), which are also used because of their bioadhesive potential [16], besides modifying the flow characteristics of gels, suspensions or emulsions. Carbomer homopolymer type A (C971) is a lightly cross-linked PAA and has shown thickening, suspending, and emulsion stabilizing properties in low-viscosity systems for topical applications [17].

Therefore, the aim of this study was to develop MTX-loaded surfactant systems composed of glycol copolymer silicone fluid (SFGC) as oil phase (OP), polyether functional siloxane (PFS) as surfactant (S) and an aqueous phase (AP) consisting of an aqueous dispersion containing 0.5% (*wt*/*wt*) of carbomer homopolymer type A (C971), and to characterize them using polarized light microscopy, small angle X ray scattering, rheological studies, and bioadhesion assays. Moreover, *in vitro* release studies of the systems were performed with a diffusion cell apparatus. Finally, the *in vitro* cytotoxicity of the systems was evaluated in order to confirm that this product offers a promising and safe nanotechnology platform for the skin administration of MTX.

## 2. Results

### 2.1. Formulation Preparation

The construction of a phase diagram is an advantageous method because it allows the visual characterization of the formulations and with this, the study of a large number of compositions, identifying experimental conditions in which it is possible to obtain nanostructured systems such as microemulsions and liquid crystals, as well as transition regions between the obtained systems. The systems were classified as isotropic transparent liquid system (ITLS) and semisolid transparent systems (SSTS). When the systems were opaque, regardless of their viscosity, they were classified as translucent dispersions (TDs). From the systems diagram, we were able to select formulations that were formed spontaneously and that presented transparency and high stability for the future incorporation of MTX.

It is noticed that at a concentration of 30% (*wt*/*wt*) of aqueous phase a liquid crystal region was formed comprising concentrations of 10% to 40% (*wt*/*wt*) oil and 30% to 60% (*wt*/*wt*) surfactant. A wide microemulsion region was obtained throughout all of the aqueous phase extension, between 60% oil phase and 80% surfactant. A small emulsion region was obtained with 30% oil phase and 50% aqueous phase and surfactant.

This diagram shows the great ability of PFS to form different surfactant systems, such as MEs and LCs. Both these systems are thermodynamically stable and can be obtained simply by mixing different proportions of aqueous phase, oil phase and surfactant, making PFS surfactant advantageous for easy and low-cost production of promising drug delivery systems for MTX.

**Figure 2:**
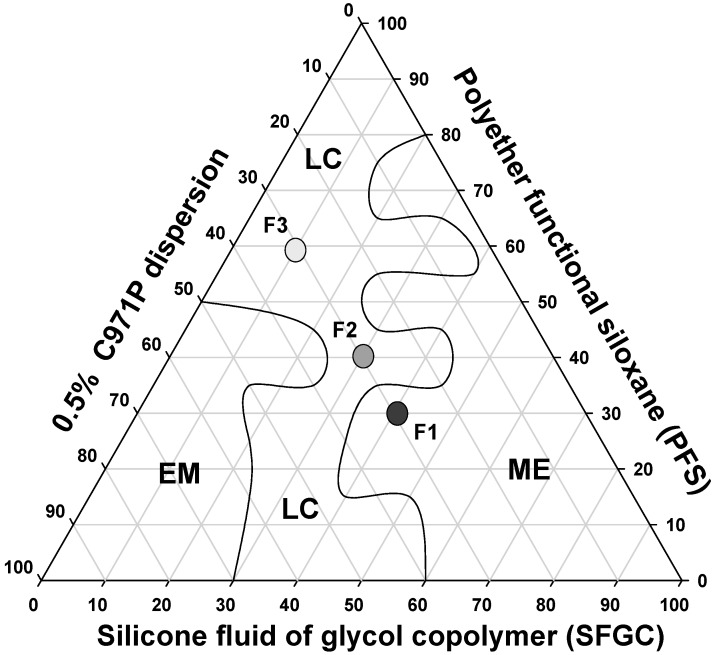
Phase diagram of system stabilized with PFS, SFGC and C971 0.5% polymeric dispersion. The marked areas represent: (LC) liquid crystal (ME) microemulsion and (EM) emulsion.

Therefore, three systems were chosen with distinct microscopic structures to analyze their rheological, bioadhesive, and mechanical properties, as well as to evaluate their capacity to control the release of MTX. The formulations chosen are indicated in the diagram in Figure 2.

### 2.2. Physicochemical and Structural Characterization of Formulations

#### 2.2.1. Phase Behavior Studies by Polarized Light Microscopy (PLM) and Small-Angle X-Ray Scattering (SAXS)

PLM can distinguish isotropic materials from anisotropic materials. Isotropic formulations, such as MEs, generate dark fields because they do not deviate the plane of light. Anisotropic formulations, such as lamellar and hexagonal LC mesophases are birefringent, so when observed by PLM, the form Maltese cross shaped structures and stretch marks, respectively. 

**Figure 3:**
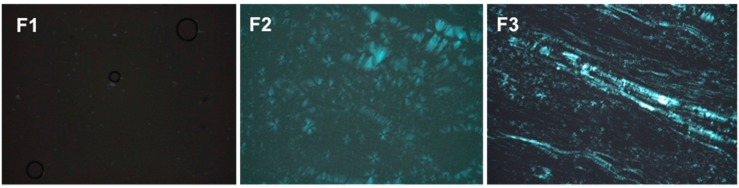
Photomicrographs obtained using polarized light microscopy (PLM) that show the dark field, the Maltese crosses characteristic of lamellar phases and the streaks of the hexagonal phases of F1, F2 and F3, respectively.

The images obtained by PLM for the three samples are shown in Figure 3. F1 presented isotropic behavior because it appeared a dark field in the photomicrographs, being therefore classified as a microemulsion. F2 was classified as a lamellar LCS, because it presented Maltese cross shaped structures. Finally, F3 was classified as a hexagonal LCS, visualized as streaks on the photomicrograph.

The structures visualized by PLM were confirmed by SAXS curves. Microemulsion systems, such as F1, show broad and undefined peaks. However, LCSs present Bragg peak intensities for specific values of the scattering vector q. Lamellar LCSs must have peaks in a ratio of 1:2:3 at the first peak, while the hexagonal liquid crystalline systems must present average distance between particle values of 1.73:2:3 [18].

From the data presented in Table 1, the structures formed by the formulations and identified by PLM were confirmed. In this way, it is suggested that the structuring of the formulation is dependent on the oil/surfactant ratio. The higher the surfactant concentration, the more liquid crystal domains are formed, making the formulation more organized [19].

In addition, data from Table 1 also show that the incorporation of MTX did not alter the phase behavior of the formulations, since the SAXS curves of unloaded and MTX-loaded formulations do not have significant differences.

#### 2.2.2. Rheology Analysis

**Figure 4:**
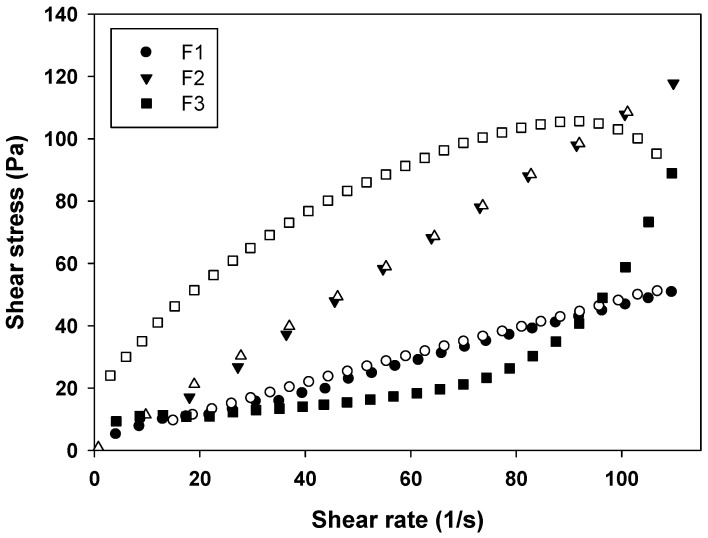
Flow rheogram of F1 (●), F2 (▼) and F3 (■). Closed symbols represents the up curve and open symbols represents the down curve. Standard deviations have been omitted for clarity; however, in all cases, the coefficient of variation of triplicate analyses was less than 10%. Data were collected at 32 ± 0.5 °C.

From the rheograms of Figure 4 and the data shown in Table 2, it is evident that the F1 behaves as a Newtonian fluid (*n* = 1), F2 behaves as pseudoplastic fluid (*n* < 1), and F3 as a dilatant fluid (*n* > 1). These results are in agreement with literature data, because MEs are known for their Newtonian behavior, while LCS phases have a non-Newtonian behavior [19].

Formulations with pseudoplastic behavior are desirable for pharmaceutical formulations, because when a stress is applied, flow thinning occurs, thus the formulation can flow easily due to breaking of the organized structures, leading to less formation of organized structures that are interesting for administrating the formulation, for example, in a wide area of the body [17,20].

In contrast, in formulations with dilatant behavior, the stress application causes a thickening of the fluid, because the particles of the formulation take on an open form upon packing. This leads to an expansion in the interparticle volume, which can form a solid paste. This is also interesting, since paste-type formulations tend to remain more time in contact with the skin releasing the drug [21].

This phenomenon demonstrated the strong influence of the LCS structure on the rheological properties of the systems, since the dilatant formulation F3 formed more organized structures when the shear rate was increased.

Furthermore, the thixotropy of the formulations was also investigated. As can be seen in Figure 4, a notable thixotropic response was only observed for F3, because the descending curve does not overlap with the ascending one, while F1 and F2 are time independent structures with overlapped upward and the downward curves due to the fast recovery of their microstructures. Thereby, a longer period of time was necessary to restore the relaxed molecular configuration, leading to the observed thixotropy.

#### 2.2.3. Texture Profile Analysis (TPA)

The mechanical properties of formulations can be defined by texture profile analysis, which are important parameters to be considered for the design of the most appropriate pharmaceutical systems. According to Table 3, formulation F3 presented the highest values for hardness, compressibility and adhesiveness. That could be due to its dilatant behavior, since the hardness and compressibility represent the work required to compress the formulation and the peak of maximum force obtained during the first compression, respectively. Consequently, both these parameters are related to the spreading of the formulation on the skin. Therefore, after the shear (compression), the dilatant F3 formulation became a formulation with more rigid structures, increasing the hardness and compressibility parameters.

In addition, the formulations with high values of hardness and compressibility tend to have high adhesiveness parameter values as well. Consequently, F3 showed the highest adhesiveness value, which was close to values seen for bioadhesive polyacrylic gels [14].

The cohesiveness values for all three formulations were not statistically different (*p* > 0.05), demonstrating that the composition of surfactant formulations did not affect the ability of the formulation to recover its initial structure.

#### 2.2.4. *In Vitro* Bioadhesion Test

**Figure 5:**
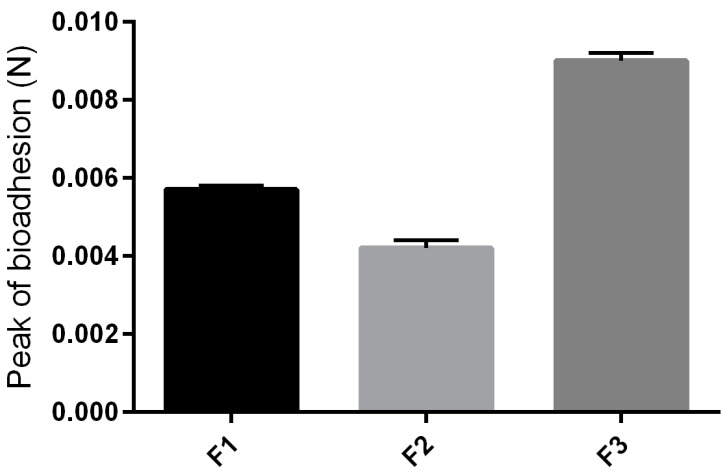
Parameters of the *in vivo* bioadhesion tests of F1, F2 and F3.

The values found for the peak bioadhesion, corresponding to the maximum force between the formulation and the skin, as a function of time force of the formulations studied are shown in Figure 5. With the addition of surfactant into the formulation, a higher consistency was obtained, and the formulation F3 revealed a greater bioadhesive force, which could improve the ability of the product to fix itself onto the skin of the user [22].

The high bioadhesion of F3 may have been influenced by its thixotrophy. Due to this characteristic, F3 takes longer to return to its less viscous original structure, so as it remains viscous for longer, it remains adhered on biological surfaces for longer too.

#### 2.2.5. *In Vitro* Release Studies

**Figure 6:**
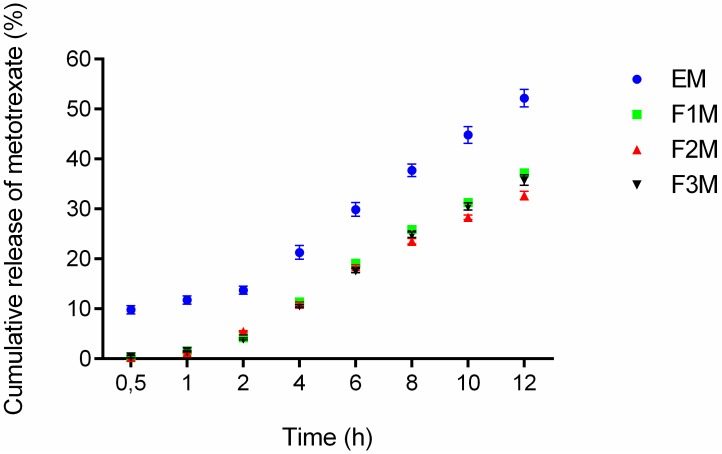
*In vitro* drug release profile of methotrexate-loaded FM1, FM2, FM3 formulations and the control EM (emulsion system) (*n* = 6).

The drug release experiments were done to verify if the formulations are capable of releasing the drug and check the amount released at a specific time interval. The formulations F1M, F2M and F3M containing 1.25% of MTX were evaluated at the following times: 0.5, 1, 2, 4, 6, 8, 10 and 12 h. According to the results after 12 h of assay 37.23% of the MTX was released from F1M, 32.68% from F2M, 35.78% from F3M, and 52.17% from the control. The observed release profiles showed that all formulations released methotrexate in a controlled manner, because an exponential pattern governed drug diffusion can be as seen in the curves of Figure 6. 

Among the three tested formulations, the formulation F1M, which has microemulsion characteristics, presented a slightly higher release percentage. This result suggests that the structure of the formulation, comprised of 30% of AP and 40% of OP, provides easier handling of the drug in the oil-water interface, being quickly available for release in the receiving solution. Moreover, the release results obtained for the F3M (hexagonal structure) and F2M (lamellar structure) formulations suggests that the structural modifications of the systems can modify the rate and profile of drug delivery [14].

Mathematical models were used to identify the mechanisms involved in MTX release. The profiles obtained in the drug delivery assay were analyzed by the models of Hixson–Crowell, Weibull, Higuchi, Baker–Lonsdale, Korsmeyer–Peppas and Hopfenberg. The adjusted coefficients of determination were used as a criteria to choose between these models. 

The measured values obtained in the drug release assay were adjusted to these mathematical models, and the Weibull model showed the highest adjusted coefficient of determination [23]. According to Weibull model, the shape parameter, *b*, characterizes the dissolution curve, indicating the release mechanism of the drug transport within the polymeric matrix. A *b* value greater than 1 indicates that the diffusion occurs by a complex release mechanism; when *b* is between 0.75 and 1 indicates a transport case II so, another mechanism has to be considered. Finally, a *b* value smaller than 0.75, means that the diffusion occurs according to Fick’s law [24]. Table 4 shows the *b* values of the analyzed formulations indicating that drug release from the formulations studied were driven by a complex release mechanism. 

This mechanism indicates that the drug release rate initially increases nonlinearly up to the inflection point, then, the release rate asymptotically decreases. Carvalho *et al.* also noted that the release profile of zidovudine (AZT) from different surfactant systems formed by polyoxypropylene (5) polyoxyethylene (20) cetyl alcohol, followed the Weibull model. However, the authors noted that the organized degree of the systems influenced the release rate, because the microemulsions follow Fickian diffusion whereas the lamellar liquid crystals show a complex mechanism. Thus, they reported that the increased amount of water in the liquid crystalline system could have enhanced the formation of hydrogen bridges between water and surfactant, therefore, contributing to the complexity of the drug release mechanism [15].

In our study, differently, the same amount of water was used in all formulations; therefore, the release of MTX appear to have been influenced only by the oil/surfactant ratio, while maintaining the same type of MTX release mechanism for the different formulations. Regarding the skin route of administration, this mechanism is very desirable for skin drug delivery systems because a more prolonged release profile across the skin may sustain the MTX concentrations for an extended time. Therefore, the results obtained in our study demonstrate that PFS systems can act as MTX skin delivery systems, contributing in the treatment and welfare of patients with psoriasis.

#### 2.2.6. *In Vitro* Cytotoxicity

**Figure 7:**
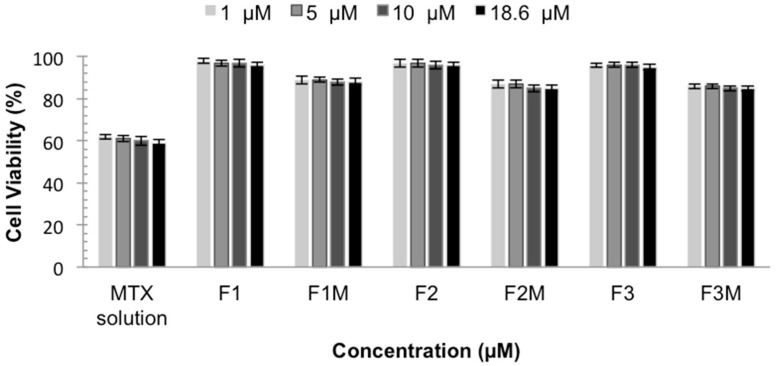
Cellular Viability of MTX solution, MTX-unloaded LCS (F1, F2 and F3) and MTX-loaded LCS (F1M, F2M and F3M) HaCaT cells for 48 h.

The *in vitro* cytotoxicity test is important to develop topical formulations with low toxicity and also to evaluate their potential to reduce the intrinsic cytotoxicity of the active compounds, such as MTX. The *in vitro* cytotoxicity tests were performed using the immortalized human keratinocyte line HaCaT as a cellular model. The data are shown as a percent of cellular viability in Figure 7.

The cell viability showed that MTX solution decreases the viability of normal keratinocytes, presenting a cellular viability around 60%. These data confirm the toxicity of MTX already described and the need to develop a system that decreases and prevents this effect [25]. It was observed that the studied formulations did not affect the viability of keratinocytes. MTX-loaded CLS also, did not show toxicity, presenting cellular viability values above 85%, indicating that the formulations are less cytotoxic to eukaryotic cells when compared to MTX solution, making them an extremely important system. Therefore, the results indicate the safety and biocompatibility of the formulations.

## 3. Materials and Methods

### 3.1. Materials

The surfactant polyether functional siloxane (PFS) is available commercially as DC^®^5329 (Dow Corning, Midland, MI, USA). Silicone glycol copolymer fluid (SFGC) is available commercially as DC^®^193 Fluid (Dow Corning), and methotrexate was acquired from Genix (São Paulo, Brazil). Carbomer homopolymer type A (C971) is available commercially as Carbopol^®^ 971P NF Polymer (Lubrizol, Cleveland, OH, USA). High-purity water was prepared with a Milli-Q plus purification system (Merck Millipore, Darmstadt, Germany), 3-(4,5-Dimethylthiazol-2-yl)-2,5-diphenyltetrazolium bromide (MTT) and phosphate buffered saline (PBS) were purchased from Sigma (St. Louis, MO, USA). Dimethyl sulfoxide (DMSO) was purchased from Viafarma (São Paulo, Brazil).

### 3.2. Preparation of Formulations

The ternary phase diagram was constructed choosing PFS as the surfactant, SFGC as the oil phase, and a dispersion of C971 at 0.5% (*wt*/*wt*) as aqueous phase. C971 was dispersed in water at 5% (*w*/*w*) and homogenized at 2000 rpm in mechanical stirrer for about 10 min. Then, the pH of C971 dispersion was adjusted with triethanolamine to 6.0 with manual agitation [14]. After this, C971 dispersion at 5% was added in each formulation to reach a final concentration at 0.5%.

At 25 ± 0.5 °C, thirty-six different proportions of 10 to 100% (*w*/*w*) of each phase formulation were weighed and mixed by hand, then left to stand for 24 h to allow the formulation to reach complete equilibrium. Each final formulation had a pH of 5.5. After that, the formulations were analyzed by polarized light microscopy as described below, and the ternary phase diagram was constructed using the Sigma Plot software (10.0, Systat Software, San Jose, CA, USA) based on the structures identified.

Afterwards, the formulations F1, F2 and F3 were selected for further physicochemical and structural characterization. Then, n amount of MTX (1.25% *wt*/*wt*) was added in the OP of F1, F2 and F3, resulting, respectively, in the formulations F1M, F2M, F3M. The component concentrations are shown in Table 5.

### 3.3. Physicochemical and Structural Characterization of Formulations

#### 3.3.1. Polarized Light Microscopy (PLM)

The samples to be examined under polarized light were prepared placing a drop of each formulation between a cover slip and a glass slide at room temperature (25 ± 0.5 °C). A BX41 polarized light microscope coupled with a QColor3 camera was used (Olympus Corporation, Tokyo, Japan) at the magnification of 20.000× to analyze the various fields of each sample.

#### 3.3.2. Small-Angle X-ray Scattering (SAXS)

SAXS data were collected at the Synchrotron SAXS beam line at the National Laboratory of Synchrotron Light (LNLS, Campinas, Brazil) at the D11A-SAXS1 station. The beam line was, equipped with an asymmetrically cut and bent Si (111) monochromator (λ = 1.608 Å) to yield a horizontally focused beam. A vertical position-sensitive X-ray detector and a multichannel analyzer were used to record the SAXS intensity, I(q), as a function of the modulus of the scattering vector q, where q = (4π/λ)sin(ε/2). In this equation, ε is the scattering angle. MTX-unloaded formulations and MTX-loaded formulations were placed in a cell at 25 °C. The parasitic scattering produced by slits was subtracted from the total scattering intensity.

#### 3.3.3. Rheology Analysis

The rheological sample analysis was performed in triplicate at 32 ± 0.1 °C, using a controlled-stress rheometer AR2000 (TA Instruments, New Castle, DE, USA) equipped with cone plate geometry (40 mm diameter) and sample gap of 52 μm. Samples of the formulations were carefully applied to the lower plate, ensuring that sample shearing was minimized, and allowed to equilibrate for 3 min prior to analysis. Continual testing was performed using a controlled shear rate procedure in the range from 0.01 to 110 s^−1^ and back, each stage lasting 120 s with an interval of 10 s between the curves. The consistency index “κ” and flow index “η” were determined using the power-law model described by the expression below where “τ” is shear stress and “γ” is shear rate [26]:

τ = κ . γ^η^ (1)

In this model, *n* > 1 represents a shear-thickening fluid; *n* < 1 represents a shear-thinning fluid and Newtonian fluids have an *n* value of unity.

#### 3.3.4. Texture Profile Analysis (TPA)

TPA was performed with a TA-XTplus texture analyzer (Stable Micro Systems, Surrey, UK). For all the formulations tested, 12 g of sample were weighed and centrifuged (Sorval TC 6 centrifuge, Du Pont, Newtown, CT, USA) to eliminate air bubbles. The sample was placed below the 10 mm analytical probe, which was lowered at a constant speed (0.5 mm/s) until it reached the sample. Contact was detected by a 2 mN triggering force and then the probe continued down to a depth 10 mm in to the sample. Next, the probe returned to the surface (0.5 mm/s) and after 5 s, a second compression started. The test provides a force-time curve from which mechanical parameters can be calculated, including hardness, compressibility, adhesiveness and cohesion. Seven replicates were analyzed at 25 ± 0.5 °C.

#### 3.3.5. *In Vitro* Bioadhesion Test

Porcine ear skin was obtained from a local slaughterhouse and prepared for the test as described by Carvalho [27]. The bioadhesive force between the pig ears’ skin and the formulations was assessed by detachment test using the TA-XTplus texture analyzer. Before the test, the skin was attached to the lower end of a cylindrical probe (diameter 10 mm) with a rubber ring. Samples of formulations were packed into shallow cylindrical vessels and the test started lowering the analytical probe, which contained the skin at a constant speed (1 mm/s) onto the surface of the sample. The skin and the sample were kept in contact during 60 s and no force was applied during this interval. After 60 s, the skin was drawn upwards (0.5 mm/s) until the contact between the surfaces was broken. The bioadhesive force of the formulations was measured in the maximum detachment force as the resistance to the withdrawal of the probe, what reflects the bioadhesion characteristic. Seven replicates were analyzed at 32 °C ± 0.5 °C.

#### 3.3.6. *In Vitro* Drug Release Study

*In vitro* release of MTX from the formulations was determined using a Franz’s cell apparatus (Hanson Research Corporation, Chatsworth, CA, USA). Synthetic cellulose acetate membrane (molar mass cut-off 12–14 kDa) with an area of 1.77 cm^2^ was previously treated with Milli-Q water for 5 min. Samples with about 300 mg of F1M, F2M, F3M and an emulsion system (EM), used as a control (30% S, 60% AP and 10% of OP), were placed on the membrane surface at the donor compartment. The latter compartment was filled with 7 mL of 0.2 M pH 7.4 phosphate buffer. The receptor solution was constantly stirred at 300 rpm and maintained at 32.0 ± 0.5 °C in sink conditions. The release samples (2 mL) were collected automatically after 30 min, 1, 2, 3, 4, 10 and 12 h, using a microette system (0700-1251, Hanson, Chatsworth, CA, USA) and replaced by same amount of fresh dissolution medium. At the end of the experiment, MTX was quantified by high performance liquid chromatography (HPLC) using the same conditions described by Von Zuben [18]. A calibration curve was constructed by preparing working solutions of MTX in 10 mM monobasic phosphate buffer (pH 7.4) at concentrations ranging from 0.05 to 150 μg/mL. The results were expressed as average of six measurements and the error was reported as standard deviation (SD).

#### 3.3.7. *In Vitro* Cytotoxicity

Cell viability tests were performed using the immortalized human keratinocyte line HaCaT as the cellular model. Cells were seeded at a density of 2.5 × 105 to 10 × 105 cells/well (96-wells plate) and cultured for 48h, exposed to different formulation doses (18.6, 10, 5 and 1 μM). After treatment, the cells were washed once with PBS, removing the formulation. Cell viability was assessed by a 3-(4,5-dimethylthiazol-2-yl)-2,5-diphenyltetrazolium bromide (MTT) reduction test, which is based on the reduction of MTT to formazan by mitochondrial dehydrogenase. Cells and MTT (0.4 mg/mL) were incubated in humidified atmosphere at 37 °C for 3 h. After the incubation period, the supernatant was removed and formazan crystals were dissolved in DMSO (180 μL). The plates were shaken for 10 min, and the optical densities were measured at 540 nm in a multiwell spectrophotometer. Each concentration was assayed three times and six additional controls (cells in medium) were used for each test. Cell viability was calculated using the following equation:

Cell viability (%) = [OD_540_ (sample)/OD_540_ (control)] × 100 (2)

### 3.4. Statistical Analysis

The data from physicochemical characterization tests, skin permeation and retention tests were evaluated by One-way ANOVA and the Tukey’s *post hoc* test with a significance level of 0.05%. Analyses were performed using the GraphPad Prism software version 5.01 (GraphPad Software, La Jolla, CA, USA, 2007).

## 4. Conclusions

This study demonstrated that the proposed systems containing polyether functional siloxane as a surfactant, silicone glycol copolymer fluid as oil phase, and an aqueous phase comprising an aqueous dispersion containing 0.5% of C971 are effective in the controlled release of MTX, presenting texture profile and bioadhesion suitable for skin use. The results showed that the structural modifications of the system could modify the rate and profile of drug delivery. The *in vitro* drug release profiles correlate well with the mathematical model used. This study revealed that MTX was released faster when incorporated in the control (emulsion) and microemulsion (F1M) system than in the LC ones (F2M and F3M), but all three formulations released the MTX in a controlled manner, since an exponential pattern governed the MTX diffusion. The *in vitro* cytotoxicity assays showed that MTX-unloaded LCS and microemulsion are not cytotoxic, therefore, the results presented here describe new skin systems for application of MTX.

## Figures and Tables

**Table 1:** Peak positions (*q*) of the SAXS curves and assignments for phase classification (d_1_/d_2_, d_1_/d_3_) of formulations F1–F3 without MTX and F1M-F3M containing MTX.

Formulation	*q*_max1_	*q*_max2_	*q*_max3_	d_1_/d_2_ (Å)	d_1_/d_3_ (Å)	Structure
F1	1.06	-	-	-	-	Microemulsion
F1M	1.07	-	-	-	-	Microemulsion
F2	0.76	1.52	2.28	2	3	Lamellar
F2M	0.79	1.58	2.33	2	3	Lamellar
F3	0.97	1.68	1.94	1.73	2.00	Hexagonal
F3M	0.96	1.67	1.94	1.73	2.02	Hexagonal

**Table 2:** Flow index (η) of F1, F2 and F3. Each value represents the mean (±standard deviation) of three replicates.

Formulations	η
F1	1.034 ± 0.094
F2	0.921 ± 0.024
F3	2.590 ± 0.292

**Table 3:** Mechanical properties of formulations with different concentrations of surfactant and oil.

Formulation	Hardness (N)	Compressibility (N∙s)	Adhesiveness (N∙s)	Cohesiveness (Dimensionless)
F1	0.019 ± 0.002	0.300 ± 0.027	0.036 ± 0.019	0.798 ± 0.048
F2	0.016 ± 0.001	0.255 ± 0.020	0.029 ± 0.026	0.800 ± 0.057
F3	0.056 ± 0.008	0.840 ± 0.008	0.766 ± 0.363	0.792 ± 0.014

Each value represents the mean (±SD) of three replicates.

**Table 4:** Estimations for R^2^ adjusted and *b* derived from fitting of equations from *in vitro* drug release data.

Formulation	Mathematical Parameters of MTX Release	
	R^2^_adjusted_	*b*
F1M	0.9994	1.36
F2M	0.9979	1.17
F3M	0.9998	1.48
EM	0.9989	2.63

**Table 5:** Rates of the components of MTX-unloaded LCSs (F1, F2, F3) and MTX-loaded LCSs (F1M, F2M, F3M). Oil phase (OP) is SFGC; aqueous phase (AP) is 5% (*wt*/*wt*) C971 dispersion; surfactant (S) is PFS.

Components	Formulations (% *w*/*w*)					
	F1	F2	F3	F1M	F2M	F3M
**Aqueous phase**	30	30	30	30	30	30
**Oil phase**	40	30	10	40	30	10
**Surfactant**	30	40	60	30	40	60
**Methotrexate**	-	-	-	1.25	1.25	1.25
**O:S ratio**	1.33	0.75	0.17	1.33	0.75	0.17
